# Microvessel ultrasound of neonatal brain parenchyma: feasibility, reproducibility, and normal imaging features by superb microvascular imaging (SMI)

**DOI:** 10.1007/s00330-018-5743-1

**Published:** 2018-10-09

**Authors:** Katharina Goeral, Azadeh Hojreh, Gregor Kasprian, Katrin Klebermass-Schrehof, Michael Weber, Christian Mitter, Angelika Berger, Daniela Prayer, Peter C. Brugger, Klara Vergesslich-Rothschild, Janina M. Patsch

**Affiliations:** 1 Department of Pediatrics and Adolescent Medicine, Division of Neonatology, Intensive Care and Pediatric Neurology, Medical University of Vienna, Waehringer Guertel 18-20, 1090 Vienna, Austria; 2 Department of Biomedical Imaging and Image-Guided Therapy, Division of General and Pediatric Radiology, Medical University of Vienna, Waehringer Guertel 18-20, 1090 Vienna, Austria; 3 Department of Biomedical Imaging and Image-Guided Therapy, Division of Neuroradiology and Muskuloskeletal Radiology, Medical University of Vienna, Waehringer Guertel 18-20, 1090 Vienna, Austria; 4 Center of Anatomy and Cell Biology, Division of Anatomy, Medical University of Vienna, Waehringer Guertel 18-20, 1090 Vienna, Austria

**Keywords:** Ultrasound imaging, Microvasculature, Neonate, Brain

## Abstract

**Objectives:**

To evaluate the feasibility and reproducibility of superb microvascular imaging (SMI) of the neonatal brain and to describe normal imaging features.

**Methods:**

We performed transcranial ultrasound with SMI in 19 healthy term-born neonates. SMI was done according to a structured examination protocol, using two linear 18 MHz and 14 MHz transducers. Superficial and deep scans were acquired in the coronal and sagittal planes, using the left and right superior frontal gyri as anatomical landmarks. All SMI views were imaged by monochrome and colour SMI and evaluated with respect to visibility of extrastriatal (i.e. cortical and medullary) and striatal microvessels.

**Results:**

We have described normal morphologic features of intraparenchymal brain microvasculature as “short parallel” cortical vessels, “smoothly curved” medullary vessels, and deep striatal vessels. In general, SMI performance was better on coronal views than on sagittal views. On superficial coronal scans, cortical microvessels were identifiable in 90–100%, medullary microvessels in 95–100%. On deep scans, cortical and medullary microvessels were visible in all cases, while striatal microvessels were identifiable in 71% of cases.

**Conclusions:**

Cerebral SMI ultrasound is feasible and well-reproducible and provides a novel non-invasive imaging tool for the assessment of intraparenchymal brain microvasculature (extrastriatal and striatal microvessels) in neonates without the use of contrast.

**Key Points:**

*• Superb microvascular imaging* (*SMI*) *of the neonatal brain is feasible and reproducible.*

*• SMI depicts extrastriatal and striatal microvessels.*

*• SMI detects two types of extrastriatal microvessels*: *cortical and medullary.*

**Electronic supplementary material:**

The online version of this article (10.1007/s00330-018-5743-1) contains supplementary material, which is available to authorized users.

## Introduction

Intraparenchymal brain vasculature consists of a hierarchical network of small arteries, veins, and capillaries. Initial anatomical descriptions date back to the mid-sixteenth century [2]. Anatomical studies addressing the macro- and micro-anatomy of the neonatal human cerebral vasculature have been conducted on post-mortem specimens and sections [3–7]. Main arterial trunks supply the cerebrum by giving rise to basal perforating arteries proximally and pial cortical arteries peripherally [3]. Pial arteries arborise into intracortical, subcortical, and medullary arteries which supply the cortex, while the cerebral white matter is exclusively supplied by medullary arteries [8–10].

Apart from invasive procedures, such as digital subtraction angiography, in vivo visualisation of neonatal cerebral vasculature is possible using computed tomography (CT) and magnetic resonance imaging (MRI). CT is limited by the use of radiation and the need for intravenous contrast material and MRI by the demanding logistics and the use of sedation, when imaging neonates. With diameters of 100–200 μm, intraparenchymal brain vessels typically remain beneath the spatial resolution of clinical imaging methods including CT and MRI (field strengths up to 3 Tesla) [11].

In neonates with patent fontanels, transcranial ultrasound is used as the first-line imaging method due to wide availability with the option of bedside use, low cost, a patient-friendly safety profile, and high image resolution [12]. Targeting blood flow in vascular structures, Doppler investigations can further enhance the clinical value of transcranial ultrasound [13].

Recently, vascular ultrasound has been given a new twist by the establishment of an innovative method referred to as “Superb microvascular imaging (SMI)” [14]. SMI has evolved as a novel tool for non-invasive imaging of microvasculature without the use of intravenous contrast material [14]. The method uses advanced clutter suppression and processes low flow Doppler signals that are otherwise filtered and removed as “clutter”. Advantages are high resolution and frame rate, and visualisation of low-velocity flow. Two modes are available: monochrome SMI (mSMI) and colour SMI (cSMI). cSMI displays low flow components in colour overlaid on the grey-scale image with high temporal and spatial resolution simultaneously. mSMI reveals microvasculature with even higher sensitivity by subtracting the anatomical background.

Ishikawa et al used SMI to image tumour vessels and tumour margins during open brain surgery in adults [15]. Regarding paediatric applications, SMI has been shown helpful in the assessment of vesico-urethral reflux [16] as well as in undescended testes [17, 18].

Currently, little is known about the in vivo morphology of the microvascular architecture of the human brain after birth. Our study aims to visualise microvessels of the neonatal brain using SMI ultrasound. Firstly, the feasibility and reproducibility of transfontanellar SMI is assessed in a cohort of term-born neonates. Secondly, normal SMI features in the healthy neonatal brain are described.

## Methods

### Study design and participants

We conducted a prospective single-centre imaging study in term-born neonates: for the main study, a total of 19 newborns were included at the Department of Paediatrics and Adolescent Medicine of the Medical University of Vienna, Austria during a 1-year study period. Inclusion criteria were defined as follows: term-born neonates with good postnatal adaptation without neurological deficits or suspected cerebral pathologies. Medical history during pregnancy had to be unremarkable, prenatal ultrasound of the central nervous system was required to be normal. Therefore, newborns with chromosomal abnormalities, postnatal neurological deficits, or any cerebral lesion diagnosed by prenatal or postnatal ultrasound were excluded from the study participation. Informed consent was obtained in all patients. The study was approved by the Ethics Committee of the Medical University of Vienna (EK 1530/2015). In order to design and plan the main study, five newborns, which were not part of the present study, underwent a structured exploratory application of SMI, which was compared with normal colour Doppler.

### Standard transcranial ultrasound

All participants underwent standard transcranial ultrasound using a Toshiba Aplio 400 scanner (Canon Medical Systems Corporation) during the first weeks of life in an awake state. A small curved array transducer (11 MHz) was placed on the patent anterior fontanel to acquire coronal and sagittal standard views according to the Austrian and German Society for Ultrasound in Medicine [19]. In addition, a mid-sagittal colour Doppler image and resistive indices were obtained from the internal carotid artery and the anterior cerebral artery. Images were stored and read using a radiologic picture archiving and communication system (PACS, AGFA HealthCare). Routine radiology reports were generated for all participants.

### SMI ultrasound—image acquisition and interpretation

**Fig. 1:**
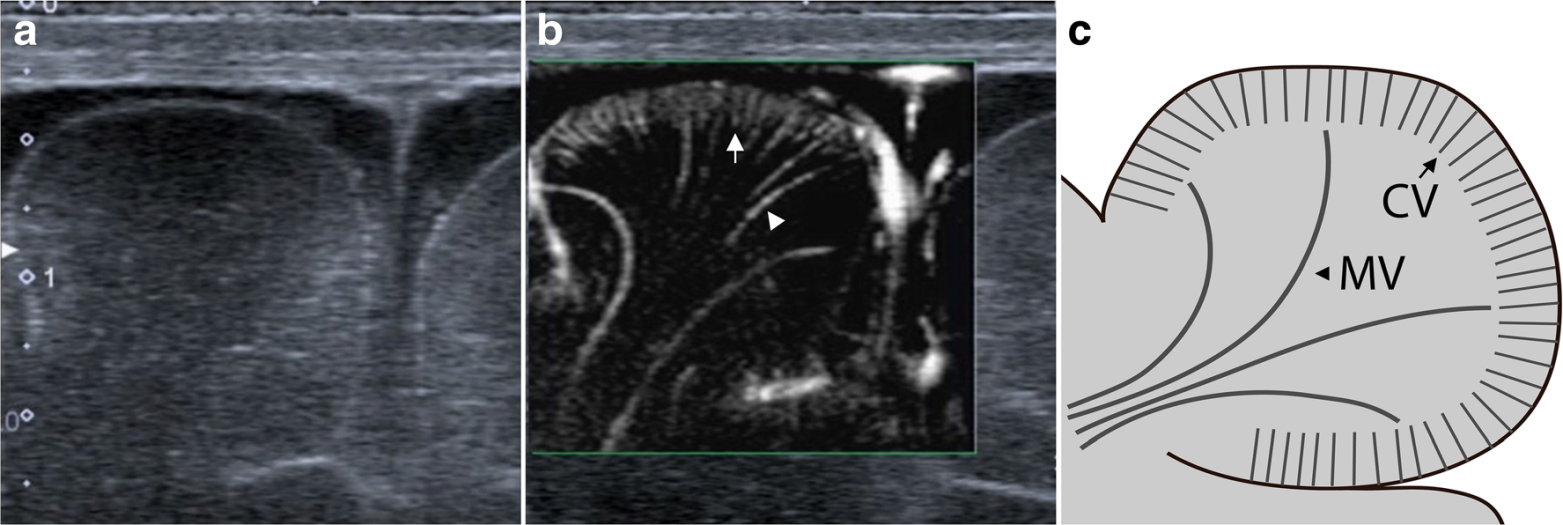
Superficial scans: Coronal view of the right superior frontal gyrus in B-mode (**a**), monochrome SMI (**b**), and schematic drawing (**c**). **b** and **c** demonstrate cortical (short arrow) and medullary (arrowhead) vessels

**Fig. 2:**
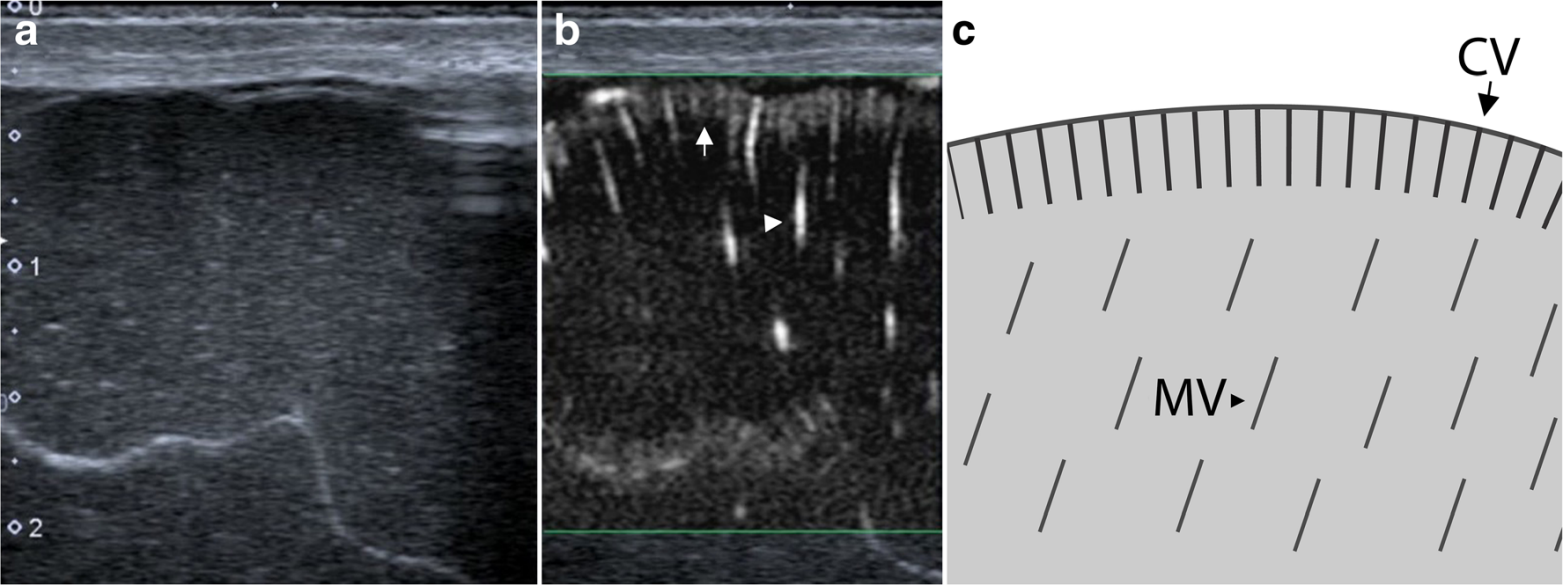
Superficial scans: Sagittal view of the right superior frontal gyrus in B-mode (**a**), monochrome SMI (**b**), and schematic drawing (**c**). **b** and **c** demonstrate cortical (short arrow) and medullary (arrowhead) vessels

**Fig. 3:**
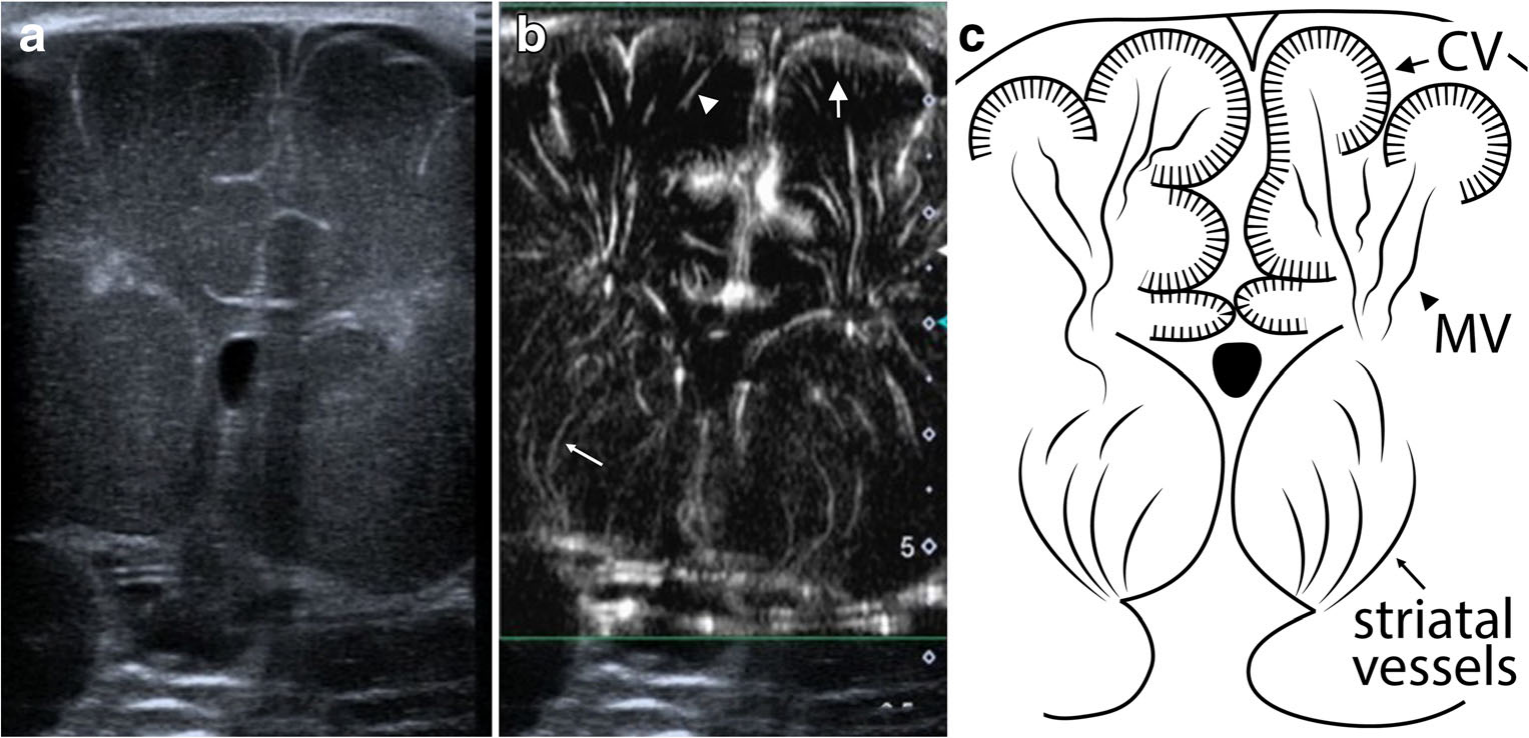
Deep scan: Coronal view in B-mode (**a**), monochrome SMI (**b**), and schematic drawing (**c**). **b** and **c** demonstrate extrastriatal (cortical [short arrow], medullary [arrowhead]) and striatal (thin arrow) vessels

SMI ultrasound was performed and documented using a predefined examination protocol by two board-certified paediatric radiologists (JP and AH), with more than 5 years of experience in neonatal head ultrasound and anatomical knowledge of the angioarchitecture of brain parenchyma. The examination protocol included superficial and deep scans. Superficial scans were acquired using a linear 18 MHz transducer (Toshiba 18L7) and captured coronal and sagittal views of the left and right superior frontal gyri (neuro-surgically abbreviated and referred to as F1 [1]; Figs. 1 and 2). F1 was chosen because of proximity to the anterior fontanel and thus optimal sonographic accessibility. Maximum zoom depth of a superficial scan was 2.5 cm. SMI box depth of superficial scan was 1.7 cm. SMI setting were 7.2 MHz (Doppler frequency), 21 kHz (Pulse repetition frequency), and colour gain 45–50. Deep scans were acquired only in the coronal plane using a linear 14 MHz (Toshiba 14L5) transducer (Fig. 3). SMI box depth of deep scans was 5–6 cm. SMI setting were 7 MHz (Doppler frequency), 9 kHz (Pulse repetition frequency), and colour gain 45–50. For a typical ultrasound exam, safety parameters were as follows: mechanical index 0.8–1.5, bone thermal index 0.6–0.8, and soft tissue thermal index 0.6–0.8.

All predefined views were imaged by monochrome and colour SMI using setting recommendations provided by the manufacturer. Grey-scale video sequences were acquired and documented twice consecutively, while colour sequences were acquired and documented once per anatomical sub-region. The minimum duration of each video sequence was 5 s. SMI video sequences were stored and reviewed using AGFA PACS. Based on radiologic feature observations made during image acquisition, a structured semi-quantitative reading scheme was developed and used (Supplementary Material): following this scheme, vessels were classified as visible or invisible. The classification and nomenclature of the angioarchitecture was adopted from Nelson et al [20] and Okudera et al [7]. Specifically, we distinguished two main vascular territories: extrastriatal vessels (supplying cortex and white matter) and striatal vessels (supplying caudate and lentiform nucleus). These vessels were rated as visible, if they appeared as bright echogenic curvilinear structures on mSMI video sequences or as red curvilinear structures on cSMI sequences.

To test inter-rater and intra-rater reproducibility, images were read off-line twice by the main reader (JP) with a time interval of 6 months between readings and once by a second reader (KG).

### Statistical analysis

Statistical analysis was performed using IBM SPSS Statistics for Windows 23.0 (IBM Corporation). Metric data are described using means ± SD if normally distributed or as median (IQR) for skewed data. Nominal data are shown as counts and percentages. Because of the exploratory character of the study, no detailed statistical tests were performed. Due to small sample size and binary data yield of SMI readings, agreement was assessed as percentage of concordat ratings instead of using Cohen’s kappa or ICC.

## Results

**Fig. 4:**
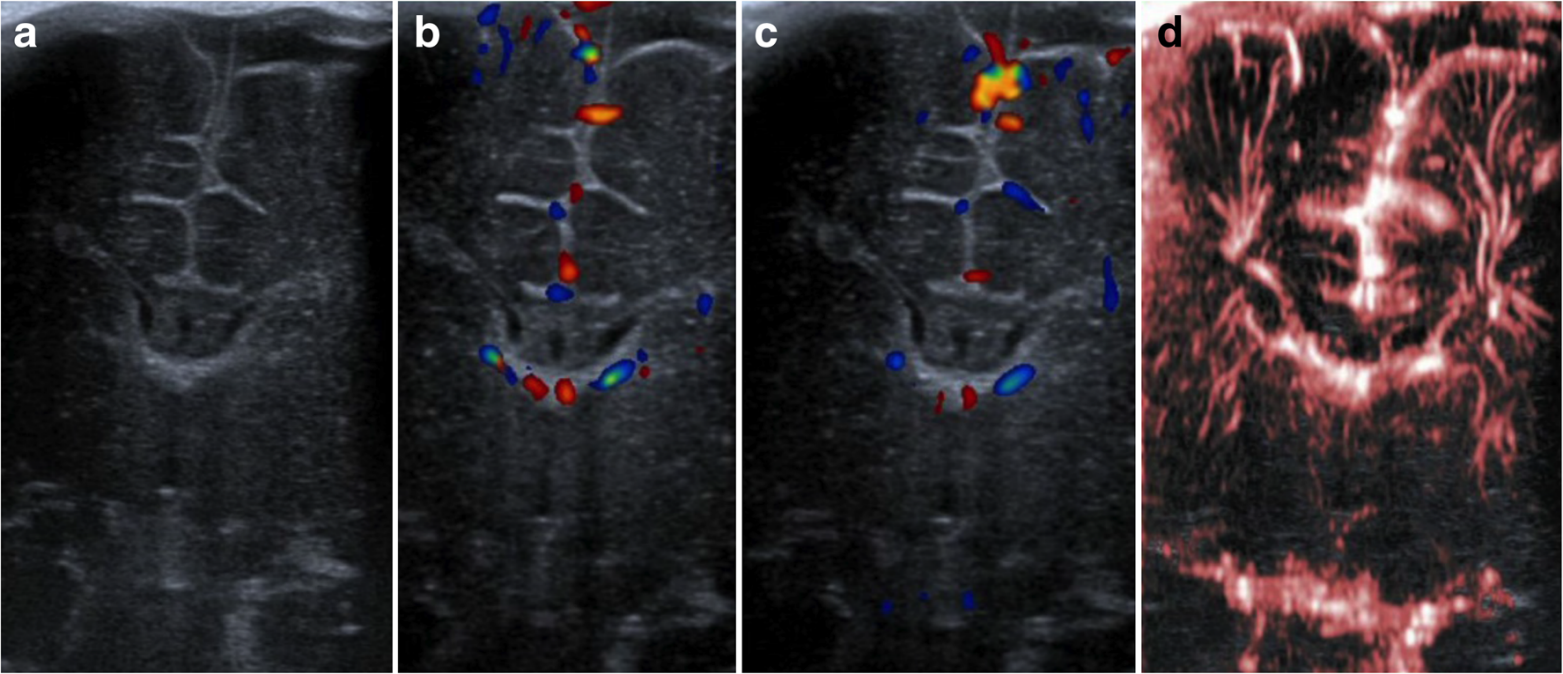
Comparison between clinical ultrasound and SMI: coronal deep view in B-mode (**a**), normal colour Doppler ultrasound (**b**, **c**), and colour SMI (**d**)

In order to design and plan the main study, five neonates underwent a structured exploratory application of SMI which was compared with normal colour Doppler ultrasound: while normal colour Doppler was able to depict some striatal and extrastriatal microvessels, there was striking superiority of SMI with regard to anatomical detail and density of visible microvessels in all five cases (Fig. 4).

For the main study, 19 term born neonates born with a mean gestational age of 38.7 weeks (± 1.5 weeks SD) were included. Newborns included in this study were mainly hospitalised due to maternal problems and not due to neonatal disease. Neonatal diagnoses included transient hypoglycaemia and mild hyperbilirubinaemia. Seventeen (17/19, 90%) were inborn, two (2/19, 11%) were outborn and were transferred to our unit for monitoring or further treatment (e.g. glucose infusion, phototherapy). SMI examination was performed at a median age of 3 days (IQR 2–4) postpartum. Further patient characteristics are provided in Table 1.

**Table 1:** Descriptive data

	Mean ± SD; median (IQR)
Gestational age (weeks)	38.74 ± 1.54
Weight (g)	2954.00 ± 539.68
Length (cm)	49.58 ± 2.78
Head circumference (cm)	34.03 ± 1.45
Percentile weight	30.16 ± 26.65
Percentile length	49.79 ± 31.33
Percentile head circumference	49.37 ± 24.39
Apgar minute 1	9 (9–9)
Apgar minute 5	10 (10–10)
Apgar minute 10	10 (10–10)
Umbilical artery pH	7.33 ± 0.06
	Count (%)
Delivered by caesarean section	12/19 (63%)
Delivered by vaginal delivery	7/19 (37%)
Female	11/19 (58%)
Male	8/19 (42%)
Singleton pregnancies	15/19 (79%)
Twins	4/9 (21%)

**Fig. 5:**
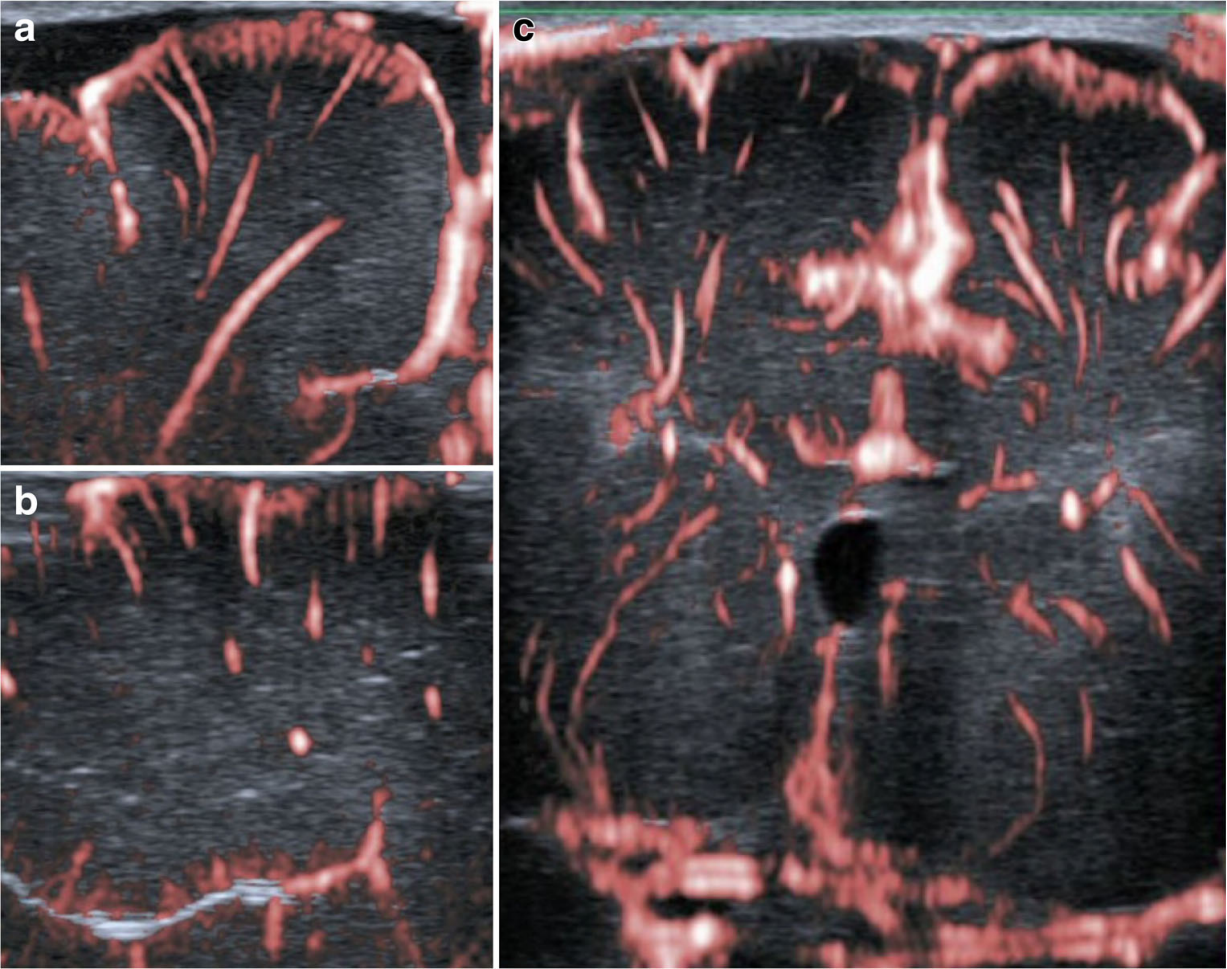
Colour SMI (cSMI): coronal (**a**), sagittal (**b**), and deep coronal (**c**) views demonstrating extrastriatal and striatal vessels superimposed to B-mode

Superficial mSMI showed extrastriatal and striatal microvessels (Figs. 1 and 2). Extrastriatal vessels were subdivided into cortical and medullary. Cortical microvessels (CV) appeared as short hyperechoic, parallel streaks perpendicular to the brain surface on coronal and sagittal views. Medullary microvessels (MV) appeared as curvilinear hyperechogenicities within the white matter displaying a characteristic “fountain-like” morphology on coronal views [7, 21]. On sagittal views, MV displayed a straight course. Deep coronal SMI showed striatal microvessels appearing as “lotus-flower” shaped curvilinear hyperechogenicities coursing through the thalamus and basal ganglia (Figs. 3 and 5).

On superficial scans, visibility of CV and MV was comparable between the left and right scan regions. In general, SMI performance was better on coronal views than on sagittal views, as both CV and MV could be identified more frequently. On sagittal views, MV were easier to visualise than CV (Table 2). On deep scans, CV and MV were visible in all cases (17/17, 100%) and striatal microvessels in 12 cases (12/17, 71%) (Tables 3 and 4). Regarding repeated monochrome acquisitions and comparability of mSMI and cSMI, visibility of MV and CV was excellent on coronal scans and lower in the sagittal plane (Table 5).

**Table 2:** Visibility of extrastriatal vessels on superficial scans

	Cortical microvessels				Medullary microvessels			
	Coronal		Sagittal		Coronal		Sagittal	
	Right F1	Left F1	Right F1	Left F1	Right F1	Left F1	Right F1	Left F1
mSMI	19/19 (100%)	18/19 (95%)	12/19 (63%)	14/19 (74%)	19/19 (100%)	19/19 (100%)	17/19 (90%)	16/19 (84%)
cSMI	17/19 (90%)	19/19 (100%)	5/17 (29%)	8/17 (47%)	18/19 (95%)	19/19 (100%)	15/17 (88%)	14/17 (82%)

*F1* superior frontal gyrus, *mSMI* monochrome SMI, *cSMI* colour SMI

**Table 3:** Inter-rater and intra-rater agreement on superficial scans

A		Cortical microvessels		Medullary microvessels	
		Coronal	Sagittal	Coronal	Sagittal
Inter-rater	1st scan	95 (90–100)	87 (84–90)	100 (100–100)	97 (95–100)
	2nd scan	97 (95–100)	97 (95–100)	100 (100–100)	97 (95–100)
	cSMI	97 (95–100)	88 (82–94)	100 (100–100)	97 (94–100)
Intra-rater	1st scan	95 (95–95)	87 (84–90)	100 (100–100)	92 (90–95)
	2nd scan	97 (95–100)	79 (79–79)	100 (100–100)	90 (90–90)
	cSMI	92 (90–95)	82 (81–82)	97 (95–100)	100 (100–100)

Data are shown as mean (min–max)

*cSMI* colour SMI

**Table 4:** Inter-rater and intra-rater agreement on deep scans

B	Cortical microvessels	Medullary microvessels	Striatal microvessels
Inter-rater	100 (100–100)	100 (100–100)	100 (100–100)
Intra-rater	94 (94–94)	100 (100–100)	88 (88–88)

Data are shown as mean (min–max)

**Table 5:** Agreement of the visibility between two monochrome acquisitions (1st and 2nd scans) and between monochrome and colour SMI

	Cortical microvessels				Medullary microvessels			
	Coronal		Sagittal		Coronal		Sagittal	
	Right F1	Left F1	Right F1	Left F1	Right F1	Left F1	Right F1	Left F1
1st and 2nd scans	19/19 (100%)	17/19 (90%)	11/19 (58%)	12/19 (63%)	19/19 (100%)	19/19 (100%)	15/19 (79%)	16/19 (84%)
mSMI and cSMI	16/19 (84%)	18/19 (95%)	5/17 (29%)	7/17 (41%)	18/19 (95%)	19/19 (100%)	15/17 (88%)	14/17 (82%)

*F1* superior frontal gyrus, *mSMI* monochrome SMI, *cSMI* colour SMI

We found excellent inter- and intra-rater agreement: both values were best for MV on coronal superficial scans and worst for CV on sagittal superficial scans (Table 3). On deep scans, concordant ratings were high for all different types of microvessels (Table 4).

## Discussion

Transfontanellar SMI ultrasound in neonates was feasible and well reproducible. From our standpoint, SMI appears to be an extremely robust and reliable technique in the setting of neonatal ultrasound. Since initial reports in March 2016 [14], SMI has been mainly used in oncologic conditions (especially breast lesions) [22–26], infectious diseases [16, 27, 28], gastrointestinal disorders [29–32], obstetric ultrasound [33, 34], musculoskeletal [35], vascular [36], and endocrine disorders [14]. In paediatric imaging, SMI has been used to detect direction of urinary flow in patients with vesico-urethral reflux without the use of contrast [16] and to demonstrate differences in vascular flow grades between normal and undescended testes, which were invisible by Power Doppler [17, 18]. Our paper is not the first reporting the use of SMI in cerebral imaging, but it is the first with a dedicated neuropediatric imaging approach. So far, only one report described the use of SMI in the adult brain: Ishikawa et al performed intra-operative ultrasound with SMI in subjects undergoing open brain surgery. They were able to recognise tumour vessels and thus to differentiate tumour from surrounding healthy tissue [15].

Our observations are in line with anatomical literature describing the morphology and course of intraparenchymal small hemispheric arteries and veins [7, 37]: vessel patterns seen in the present study are highly reminiscent of those found in neonatal brain specimens. Others have used high-resolution brain ultrasound, specifically ultrafast Doppler for in vivo assessment of cerebral hemodynamics: Demené et al have shown similar microvascular anatomy but have focused on functional imaging based on neurovascular coupling. They were able to show subtle variation in local and global brain perfusion during different sleep stages and epileptic activity [38, 39]. As opposed to them, we are using SMI, a vendor-developed tool.

In order to be consistent with existing terminology, we have decided to use the neuro-anatomic nomenclature of Nelson et al [20] and Okudera et al [7], which only differentiates two main intraparenchymal microvascular territories: striatal and extrastriatal vessels. Based on strikingly distinct SMI morphology, we have further subclassified extrastriatal microvessels as cortical and medullary.

Although SMI depicted intraparenchymal microvasculature at extraordinary detail, neither monochrome nor colour SMI could differentiate arterial from venous microvessels. Vessel characterisation by spectral Doppler had not been defined as a priori-aim of the current study. Nevertheless, we have been able to obtain characteristic arterial and venous signals in extrastriatal, specifically MV in some cases (not shown). Based on the clear superiority of SMI to normal colour Doppler ultrasound (Fig. 4), we opted against a comparative study design.

We found coronal SMI views easier to be obtained and read than sagittal views. Moreover, microvessels were better depicted on coronal views, which might be explained by the intrinsic three-dimensional anatomy of cerebral microvasculature [5, 40]. Comparing the performance of monochrome and colour SMI, our data suggest that mSMI was slightly superior to cSMI with regard to depiction of extrastriatal microvessels and image/data reproducibility. Only at a single assessment region, cSMI improved overall SMI performance. Superior performance of mSMI is most likely explained by methodical reasons: cSMI is technically optimised for the suppression of motion artefacts (both from patient and/or probe) and displays flow components in low velocity ranges in colour overlaid on the grey-scale image with high temporal and spatial resolution simultaneously. mSMI is further optimised for the detection of low flow: within the region of interest, B-mode (i.e. anatomical background) is removed from the image, resulting in high sensitivity for the depiction of microvasculature. Although we found mSMI generally superior to cSMI, cSMI might still be a relevant add-on for visual display.

**Fig. 6:**
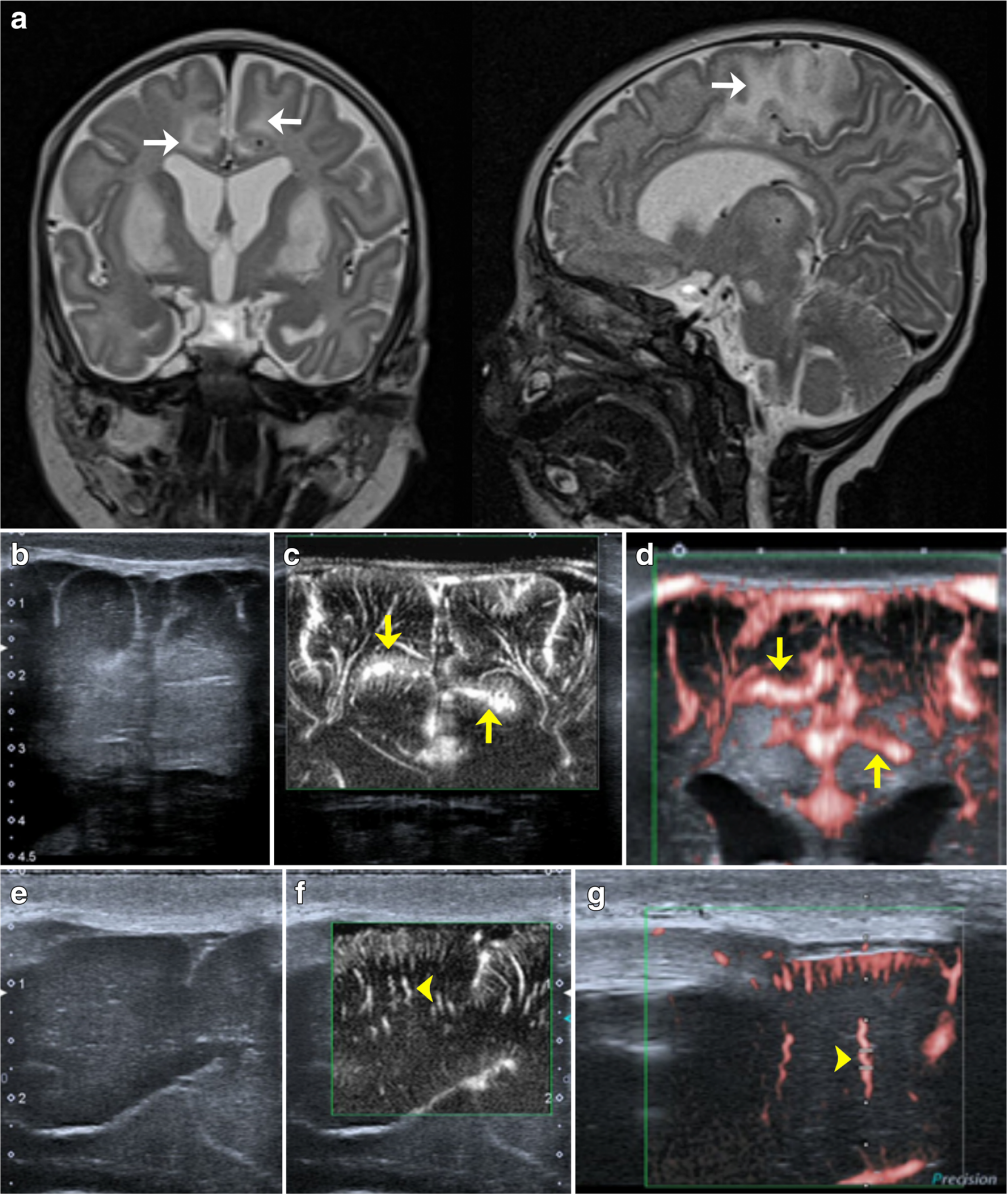
Pathologic example: 3-month old patient with mitochondrial disease (Leigh disease) imaged by MRI (**a**), routine ultrasound (**b** and **e**), and SMI (**c**, **d**, **f**, and **g**). While MRI showed metabolic infarcts and signal alterations of the basal ganglia and cortex (**a**; white arrow), coronal mSMI showed posthypoxic cortical hyperperfusion (**c** and **d**; yellow arrow). In addition, SMI showed elongated and tortuous medullary microvessels (**f** and **g**; yellow arrowhead), which were confirmed by histology (not shown)

From a clinical standpoint, it should be stressed that there is great potential for the use of SMI in the neonatal brain: hypoxic ischemic encephalopathy, cerebral malformations, infections, and preterm birth are conditions associated with microvascular abnormalities. After ischemia, SMI might allow the identification and bedside monitoring of posthypoxic hyperperfusion (Fig. 6) [6, 41]. In preterm neonates with intraventricular haemorrhage, SMI might allow the early identification of venous congestion and periventricular infarction following an intraventricular haemorrhage. In combination with neurophysiological methods, the right timepoint for pressure-reducing interventions could be identified in the presence of post-haemorrhagic hydrocephalus.

The comparison between SMI and MRI as clinical gold standard is warranted in future studies. Bearing in mind that the development of intraparenchymal brain vasculature is a continuous process starting at embryonal stages of human life and continuing throughout infancy [42], longitudinal SMI studies including preterm infants are needed.

Our study has several limitations: the sample size was small. From a practical perspective, it should be mentioned that the size of the anterior fontanel is an important limiting factor for the diagnostic quality of transcranial ultrasound. Nevertheless, in our study cohort, no patient had to be excluded due to small anterior fontanel or bad quality images. Spontaneous movements represent another limiting factor to the feasibility of cerebral SMI in newborns. We have solved this issue by instructing the parent/accompanying nurse to gently stabilise the head in a neutral position with both hands on either side, by the use of a pacifier and/or oral sucrose, and by having two operators perform the exam: one focused on image acquisition, while the other was responsible for parameter setting and documentation according to the study protocol. We are aware that in daily practise, a single operator is most likely to be performing the exam but opted for this strict approach to ensure high standards in initial reporting.

In summary, we have demonstrated practical feasibility and excellent reproducibility of transfontanellar cerebral SMI ultrasound in healthy term-born neonates. Using superficial and deep scanning modes, we have described normal morphologic features of intraparenchymal brain microvasculature as “short parallel” cortical vessels, “smoothly curved” medullary vessels, and deep striatal vessels. We conclude from our study that SMI ultrasound provides a novel non-invasive imaging tool for the assessment of intraparenchymal brain vasculature in neonates without the use of contrast material.

## Electronic supplementary material

ESM 1 (DOCX 35721 kb)

## Abbreviations

cSMI: Colour SMI
CT: Computed tomography
CV: Cortical microvessels
F1: Superior frontal gyrus (surgically referred to as F1) [1]
MRI: Magnetic resonance imaging
mSMI: Monochrome SMI
MV: Medullary microvessels
SMI: Superb microvascular imaging
