# Supplementary figures and images for: Microvessel ultrasound of neonatal brain parenchyma: feasibility, reproducibility, and normal imaging features by superb microvascular imaging (SMI)
Source: Eur Radiol. 2018 Oct 9;29(4):2127–36. doi: 10.1007/s00330-018-5743-1 (PMC6420458; doi:10.1007/s00330-018-5743-1)

**Supplementary Material.** Reading Scheme


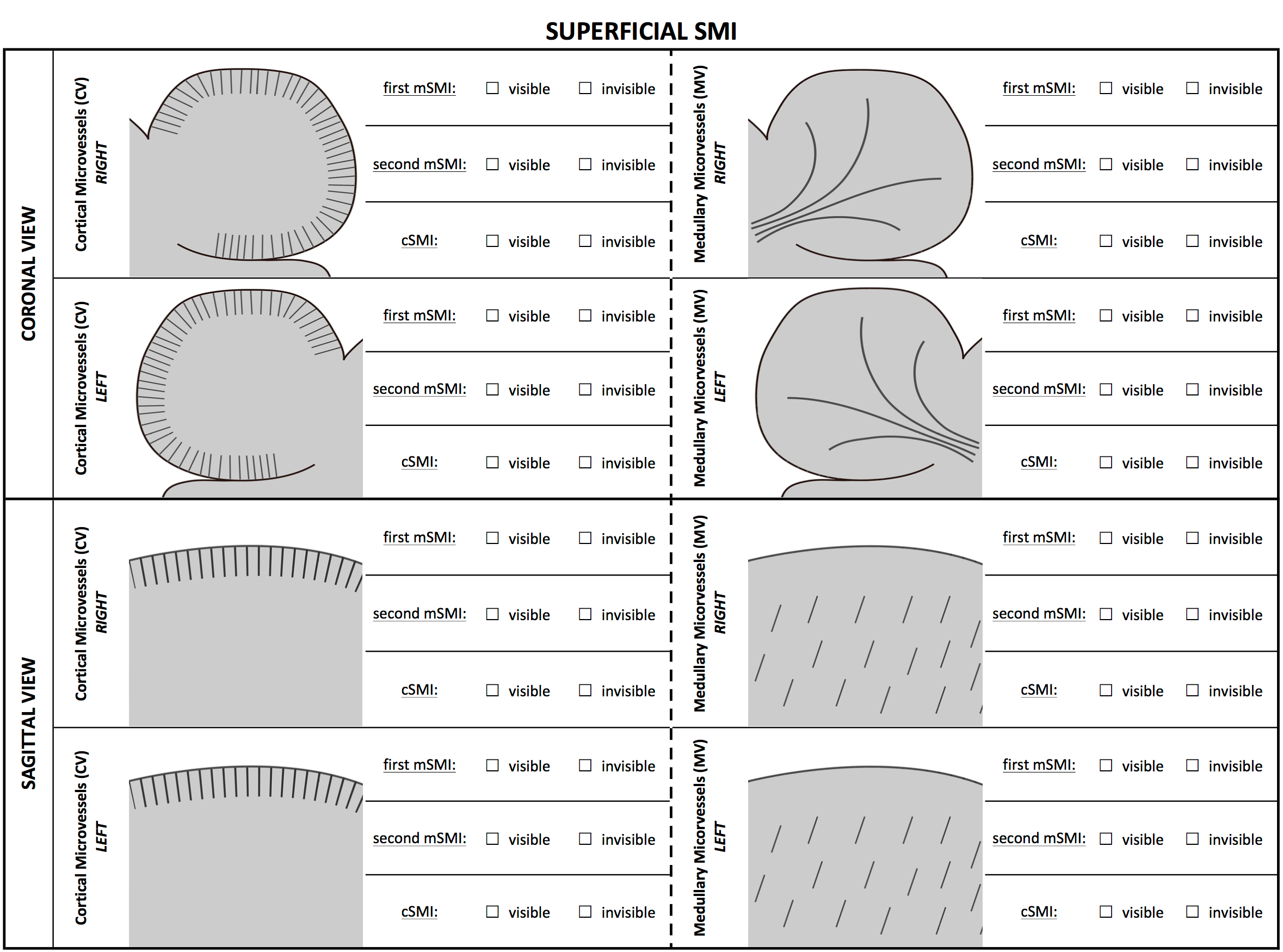


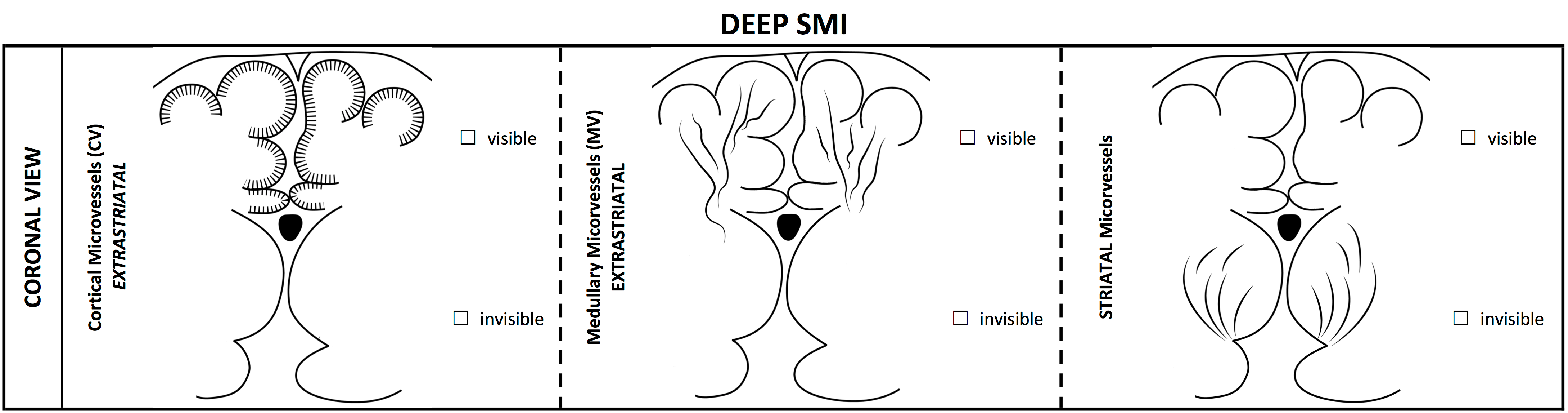

Supplement: Supplementary file 1 — (DOCX 35721 kb) [file 330_2018_5743_MOESM1_ESM.docx]
